# Down-regulation of long noncoding RNA HULC inhibits the inflammatory response in ankylosing spondylitis by reducing miR-556-5p-mediated YAP1 expression

**DOI:** 10.1186/s13018-023-04003-0

**Published:** 2023-07-31

**Authors:** LuLu Yi, ChangJun Song, YuanYuan Liu, DongSheng Li, TianTian Xiao, XuQin Guo, YiCai Wu

**Affiliations:** 1 Department of Rheumatology, Ganzhou People’s Hospital, Ganzhou City, 341000 Jiangxi Province China; 2 Department of Emergency, Ganzhou People’s Hospital, Ganzhou City, 341000 Jiangxi Province China; 3 Department of Obstetrics, Ganzhou People’s Hospital, Ganzhou City, 341000 Jiangxi Province China; 4 Department of Nephrology, Ganzhou People’s Hospital, No. 17, Hongqi Avenue, Zhanggong District, Ganzhou City, 341000 Jiangxi Province China

**Keywords:** Long noncoding RNA HULC, MiR-556-5p, YAP1, Ankylosing spondylitis, Cell proliferation, Apoptosis, Inflammatory response

## Abstract

**Objective:**

Ankylosing spondylitis (AS) is a progressive systemic disease characterized by a chronic inflammatory response in the sacroiliac joints and spine. Long noncoding RNAs suggest significant actions in the progression of AS. Therefore, a specific lncRNA, highly upregulated in liver cancer (HULC), was studied here regarding its functions and related mechanisms in AS.

**Methods:**

Measurements of miR-556-5p, HULC, and YAP1 expression were performed on AS cartilage tissues and chondrocytes. The interaction between miR-556-5p and HULC or YAP1 was verified. CCK-8, flow cytometry and enzyme-linked immunosorbent assay were used to evaluate the effects of HULC, miR-556-5p, and YAP1 on the proliferation, apoptosis, and inflammatory response of AS chondrocytes. Furthermore, the action of HULC/miR-556-5p/YAP1 was experimentally observed in AS mice.

**Results:**

HULC and YAP1 levels were augmented, while miR-556-5p levels were suppressed in AS cartilage tissues and chondrocytes. Downregulating HULC or upregulating miR-556-5p stimulated chondrocyte proliferation and inhibited apoptosis and inflammation in AS. miR-556-5p was a downstream factor of HULC, and YAP1 was a potential target of miR-556-5p. The improvement effect of downregulated HULC on AS chondrocytes was saved when YAP1 expression was forced. In addition, silence of HULC improved the pathological injury of spinal cartilage in AS mice by enhancing miR-556-5p-related regulation of YAP1.

**Conclusion:**

HULC inhibition relieves the inflammatory response in AS by reducing miR-556-5p-mediated YAP1 expression.

## Introduction

Ankylosing spondylitis (AS) is a chronic autoimmune disease characterized by systemic inflammation and pathological osteogenesis, which involves the spine and sacroiliac joints and eventually leads to the ossification of ligament and spinal fusion [1–4]. There are few cases of surgical treatment in clinical application due to high perioperative risk, cost, difficulty in operation and poor prognosis [5, 6]. Considering the adverse effects, such as increased risk of cardiovascular disease, non-steroidal anti-inflammatory drugs and anti-tumor necrosis factor-α therapy are not approved for long-term treatment of AS [7–11]. Although studies have confirmed that the etiology and pathogenesis of AS are related to bacterial infection [12, 13], macrophage activation [14], HLA-B27 misfolding [15], autophagy [16, 17], and cytokines [18, 19], the underlying mechanism of inflammatory response has not been fully elucidated. Therefore, a comprehensive insight into the molecular mechanism of the inflammation progression in AS is urgent at present.

As non-coding regulatory RNAs, long noncoding RNAs (lncRNAs) can modulate many biological processes, such as cell proliferation, apoptosis, and the release of proinflammatory cytokines [20, 21]. Till now, lncRNAs have been believed to actively involve in orthopedic diseases, including scoliosis, disc herniation, arthritis, and AS [22–24] and are considered with potential treatment effects. For example, lncRNA NKILA affects plasma TGF-β1 level [25], and lncRNA H19 mediates the release of inflammatory cytokines IL-17/IL-23 in AS [26]. Down-regulating lncRNA HOTTIP improves AS progression by inhibiting the proliferation and differentiation of fibroblast-like synovial cells [27]. Wang et al. [28] have demonstrated that lncRNA HULC promotes inflammatory responses in cholangiocarcinoma by affecting the production of IL-6. In addition, HULC exerts a regulatory role in the TNFα-induced apoptosis of human vascular endothelial cells [29]. However, HULC involved in the regulation of inflammatory response has not been studied in AS.

The competing endogenous RNA hypothesis has been recognized and confirmed in a variety of human diseases [30, 31]. Multiple studies have confirmed the role of miRNAs and mRNAs in musculoskeletal disorders [32–35]. The current paper predicted HULC-related miRNA and mRNA target through bioinformatics, and screened out miR-556-5p and YAP1. miR-556-5p, a small non-coding RNA, can mediate inflammatory responses [36, 37]. YAP1 is identified as a transcriptional coactivator that suggests modulatory roles in orthopedic degenerative diseases and affects activities of chondrocytes [38, 39].

The study aimed at discussing the interrelationship between HULC, miR-556-5p, and YAP1, and was based on the assumption that HULC can absorb miR-556-5p to regulate YAP1, and then affect the inflammatory response of AS.

## Methods

### Research objects

From 20 AS patients (15 males, 5 females; Age ranged from 21 to 45 years [mean age, 28 years]), AS cartilage tissue was collected from the hip joint. Control specimens (13 males, 7 females; Age ranged from 22 to 40, [mean age, 30 years]) were separated from 20 cases undergoing traumatic femoral head fracture or hip fracture surgery. Specimens were fixed in 4% paraformaldehyde for 2–4 h at 4 °C, placed overnight at 4 °C in PBS-30% sucrose, and stored at − 80 °C. Patients were diagnosed according to AS classification criteria (the Assessment of SpondyloArthritis international Society). Written informed consent was collected from all subjects. This study was approved by the Ethics Committee of Ganzhou People’s Hospital.

### Chondrocyte isolation and culture

Patients’ cartilage tissue was transferred to two separate Petri dishes under sterile conditions, cut into about 1-mm^3^, and collected after centrifugation. Then, the samples after detachment with 0.25% trypsin for 5 min were treated with centrifugation and resuspended in 10 ml PBS. The precipitate was added to 10 ml DMEM/F12 containing 0.2% collagenase II to incubate for 12 h under normal conditions. The cell suspension was filtered through a 200 mesh filter and collected by centrifugation, and the cell precipitate was resuspended in 10% FBS-DMEM/F12 medium to reach 1 × 10^6^ cells/ml concentration, and standard-cultured in 100-mm dishes to greater than 90% confluence.

To identify chondrocytes, chondrocytes fixed in 4% paraformaldehyde were stained with 1% toluidine blue for 30 min, followed by successive treatments with double water and absolute ethanol before microscopic examination.

### Chondrocyte transfection

Chondrocytes were seeded in 6-well plates and transfected following the protocol of Lipofectamine 2000 (Thermo Fisher Scientific, Waltham, MA, USA). The plasmids (Genechem, Shanghai, China) included HULC low expression negative control (sh-NC), HULC low expression vector (sh-HULC), HULC high expression negative control (oe-NC), HULC high expression vector (oe-HULC), mimic NC, miR-556-5p mimic, inhibitor NC, miR-556-5p inhibitor, YAP1 high expression negative control (pcDNA-NC), and YAP1 high expression vector (pcDNA-YAP1).

### RT-qPCR

Total RNA was extracted from 1 × 10^6^ cells or 0.1 g tissue using TRIzol reagent (Invitrogen, Carlsbad, CA, USA). The quality and quantity of RNA samples were evaluated by standard spectrophotometry and electrophoresis. cDNA was synthesized by PrimeScript RT Reagent Kit (Takara, Japan) using total RNA 1 μg as template. cDNA was subjected to PCR amplification: Denaturation at 92 °C for 10 s, annealing at 55 °C for 20 s and extension at 68 °C for 20 s (40 cycles). PCR was performed in a reaction mix of SYBR Green (Takara) with a CFX96 Touch Real-Time PCR Detection System (Bio-Rad, Hercules, CA, USA). U6 and GAPDH were loading controls. Table 1 exhibits sequences of gene primers.

**Table 1:** PT-qPCR primers

Genes	PCR primer sequences (5’–3’)
HULC	Forward: TCATGATGGAATTGGAGCCTT
	Reverse: CTCTTCCTGGCTTGCAGATTG
miR-556-5p	Forward: GATGAGCTCATTGTAAT
	Reverse: GCAGGGTCCGAGGTATTC
YAP1	Forward: CCCTCGTTTTGCCATGAACC
	Reverse: GTTGCTGCTGGTTGGAGTTG
U6	Forward: CTCGCTTCGGCAGCACA
	Reverse: AACGCTTCACGAATTTGCGT
GAPDH	Forward: CACCCACTCCTCCACCTTTG
	Reverse: CCACCACCCTGTTGCTGTAG

*HULC* Long noncoding RNA highly upregulated in liver cancer, *miR-556-5p* microRNA-556-5p, *YAP1* Yes-associated protein 1, *GAPDH* Glyceraldehyde-3-phosphate dehydrogenase

### Western blot

Total protein was extracted using a radioimmunoprecipitation assay kit (Beyotime, Shanghai, China), and protein concentration was determined using a BCA kit (Beyotime). Equal amounts of protein were separated by loading onto 10% SDS-PAGE, then the proteins were transferred to polyvinylidene difluoride membranes, blocked with 5% nonfat milk for 1 h, mixed with primary antibodies anti-YAP1 (ab52771; 1:1000, Abcam) and anti-GAPDH (ab8245; 1:1000, Abcam) overnight at 4 °C, and combined with goat anti-Rabbit IgG-HRP (ab205718; 1:2000, Abcam) for 2 h. Protein bands were colorized with enhanced chemiluminescence reagent (Beyotime) and evaluated by Image J software.

#### ELISA

IL-1β, IL-6, and TNF-α were detected by corresponding ELISA kits (Hengyuan, Shanghai, China). OD values at 450 nm were measured by a microplate reader (Thermo Fisher Scientific).

#### CCK-8

Chondrocyte proliferation was determined using the CCK-8 kit (Solarbio, Beijing, China). Chondrocytes cultured in 96-well plates (5 × 10^3^ cells/well) for 3 d were added with CCK-8 reagent (10 μl/well) for 2 h, and tested on a microplate reader (Thermo Fisher Scientific) to record OD values at 450 nm.

### Flow cytometry

Chondrocytes (1 × 10^6^) were stained using the FITC-Annexin V Apoptosis Detection Kit (BD Biosciences, San Jose, CA, USA) according to the manufacturer's instructions and assessed on a BD FACSCalibur flow cytometer (BD Biosciences).

### TUNEL staining

Chondrocyte slides were fixed in 4% paraformaldehyde for 30 min and immersed in 1% Triton X-100 for 3 min. Cell slides were reacted with 50 μl TdT solution for 1 h and incubated with 50 μl streptavidin-TrITC solution for 30 min. After counterstaining with 6-diamamidine-2-phenylindole for 10 min, slides were sealed with diluted glycerol and photographed under a fluorescence microscope (Olympus, Tokyo, Japan) with an excitation wavelength of 543 nm and an emission wavelength of 571 nm.

### Dual-luciferase reporter assay

The reporter vector pmirGLO-HULC-WT (wild type) was formed by cloning HULC cDNA, which contains the predictive binding site of miR-556-5p, into the pmirGLO Dual-Luciferase miRNA Target Expression Vector (Promega, Madison, WI, USA). The vector pmirGLO-HULC-MUT (mutant) was inserted by the mutant HULC, which contains point mutations of the miR-556-5p seed region binding site. Likewise, YAP1 3’UTR sequences containing wild-type or mutant miR-556-5p binding sites were inserted into pmirGLO vector (Promega). Luciferase reporters HULC-WT, HULC-MUT, YAP1-WT, and YAP1-MUT were obtained. The above plasmids and miR-556-5p mimic or mimic NC were transfected into 293 T cells using Lipofectamine 2000 (Thermo Fisher Scientific) and analyzed for luciferase activity after 48 h by dual luciferase reporter assay kit (Promega).

### RNA immunoprecipitation (RIP) assay

293 T cells were transfected with miR-556-5p mimic or mimic NC, respectively. After 48 h, RIP assays were performed on the cells based on Magna RIP™ Kit (Millipore, Bedford, MA, USA). Then, 293 T cells were incubated with anti-Ago2 (Millipore) or IgG (Millipore), and the relative enrichment of HULC and YAP1 were measured by RT-qPCR.

### Animal model

A total of 36 healthy BALB/c male mice (18–22 g, 4–5 weeks old) were maintained in stable animal experimental cages for one week. With 6 mice as normal controls, 30 mice were modeled AS by intraperitoneal injection with 0.15 mL emulsion (75 μg proteoglycan) and an additional injection after 7 d. After 21 d, joint swelling and joint pathology were evaluated.

### Grouping of experimental animals

50 mM of sh-NC, sh-HULC, oe-NC, or oe-HULC were mixed with Invivofectamine (IVF3005; Thermo Fisher Scientific) and then injected into mice via tail vein. Mice were divided into normal group (normal mice did not receive any treatment), AS group (AS mice did not receive any treatment), AS + sh-NC group (AS mice intravenously injected with sh-NC), AS + sh-HULC group (AS mice intravenously injected with sh-HULC), AS + oe-NC group (AS mice intravenously injected with oe-NC), and AS + oe-HULC group (AS mice were intravenously injected with oe-HULC), with 6 mice in each group. Mice were injected every 3 days and euthanized on day 14. Next, the mouse spine was isolated under a stereoscopic microscope, the cervical disc was removed, the vertebral cartilage endplate was separated, with attached red blood cells, bone tissue, and disc fibrous tissue removed. Subsequently, cartilage tissue was dissected into 5 mm × 5 mm, treated with liquid nitrogen, and stored at − 80 °C.

### HE staining

Cartilage tissues were prepared as paraffin-embedded blocks and sectioned into 5 μm. Then, the sections were dewaxed with xylene, dehydrated in gradient ethanol, stained with hematoxylin for 7 min, and rinsed under running water. After removing the excess dye solution, sections were dehydrated with 95% ethanol for 5 s and stained with eosin for 1 min for observations under a light microscope (Nikon, Japan).

### Safranin O staining

Sections were dewaxed with xylene, dehydrated with gradient ethanol, and rinsed with distilled water. Sections were stained with Weigert Iron Hematoxylin for 10 min and with 0.25% Fast Green Stain for 5 min and rinsed directly in 1% acetic acid. Subsequently, sections were dehydrated with gradient ethanol and stained with 1% saffron O. Finally, xylene-washed sections were observed under a light microscope (Nikon).

### Statistical analysis

SPSS 25.0 software was utilized for statistical analysis. All data are expressed as mean ± standard deviation and assessments were carried out using student's t test or one-way analysis of variance, followed by Tukey's post-hoc test. *P* < 0.05 indicates statistical significance.

## Results

### Forced expression of HULC in AS cartilage tissues and chondrocytes

**Fig. 1:**
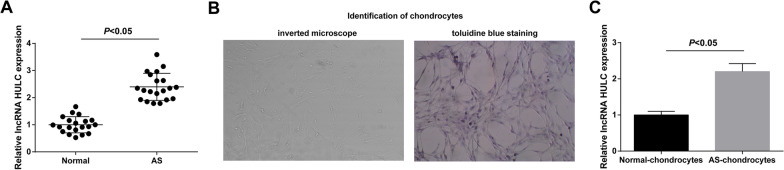
Forced expression of HULC in AS cartilage tissues and chondrocytes. HULC expression in cartilage tissue of AS patients (**A**). Identification of chondrocytes by inverted microscope and toluidine blue staining (**B**). HULC expression in AS chondrocytes (**C**). Values were expressed as mean ± standard deviation, *P* < 0.05

The abnormally high expression of HULC in cartilage tissues of AS was confirmed by RT-qPCR (Fig. 1A). Subsequently, chondrocytes were identified, and the results showed that the appearance of chondrocytes was polygonal, with abundant cytoplasm, clear nuclei, round or oval nuclei located in the center of the cell body, and 1–3 nucleoli. The cells showed good refraction and optimal colony growth (Fig. 1B). In addition, RT-qPCR results supported that HULC expression was increased in AS chondrocytes (Fig. 1C).

### Silencing of HULC promotes chondrocyte proliferation and reduces apoptosis and inflammation in AS

**Fig. 2:**
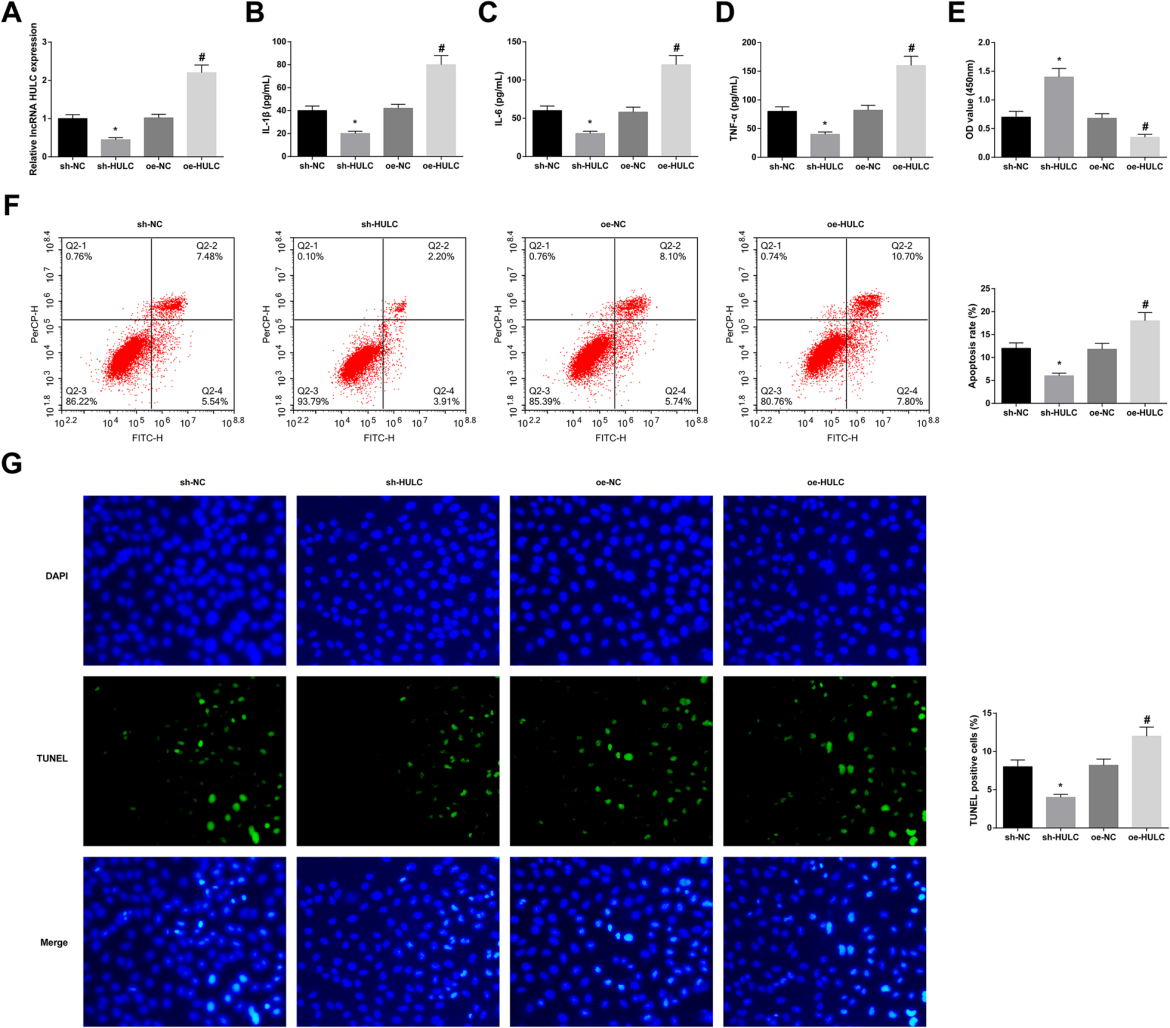
HULC affects chondrocyte proliferation and reduces apoptosis and inflammation in AS. Successful cell transfection to alter HULC expression in AS chondrocytes (**A**). After transfection, measurements of IL-1β, IL-6 and TNF-α levels (**B**–**D**), proliferation (**E**), and apoptosis (**F**, **G**). Values are expressed as mean ± standard deviation. *P < 0.05 versus sh-NC; #*P* < 0.05 versus oe-NC

RT-qPCR demonstrated that HULC expression was decreased after HULC knockdown, while it was increased after HULC overexpression (Fig. 2A). Then, IL-1β, IL-6, and TNF-α were detected by ELISA, and the results demonstrated that the three were decreased after downregulating HULC, while upregulating HULC caused the opposite trend (Fig. 2B–D). In addition, chondrocyte proliferation was checked by CCK-8, and apoptosis was by flow cytometry and TUNEL staining. The collected data exhibited that HULC silencing promoted proliferation and limited apoptosis in AS chondrocytes, while HULC up-regulation promoted the opposite trend (Fig. 2E–G).

### miR-556-5p is a downstream factor of HULC

**Fig. 3:**
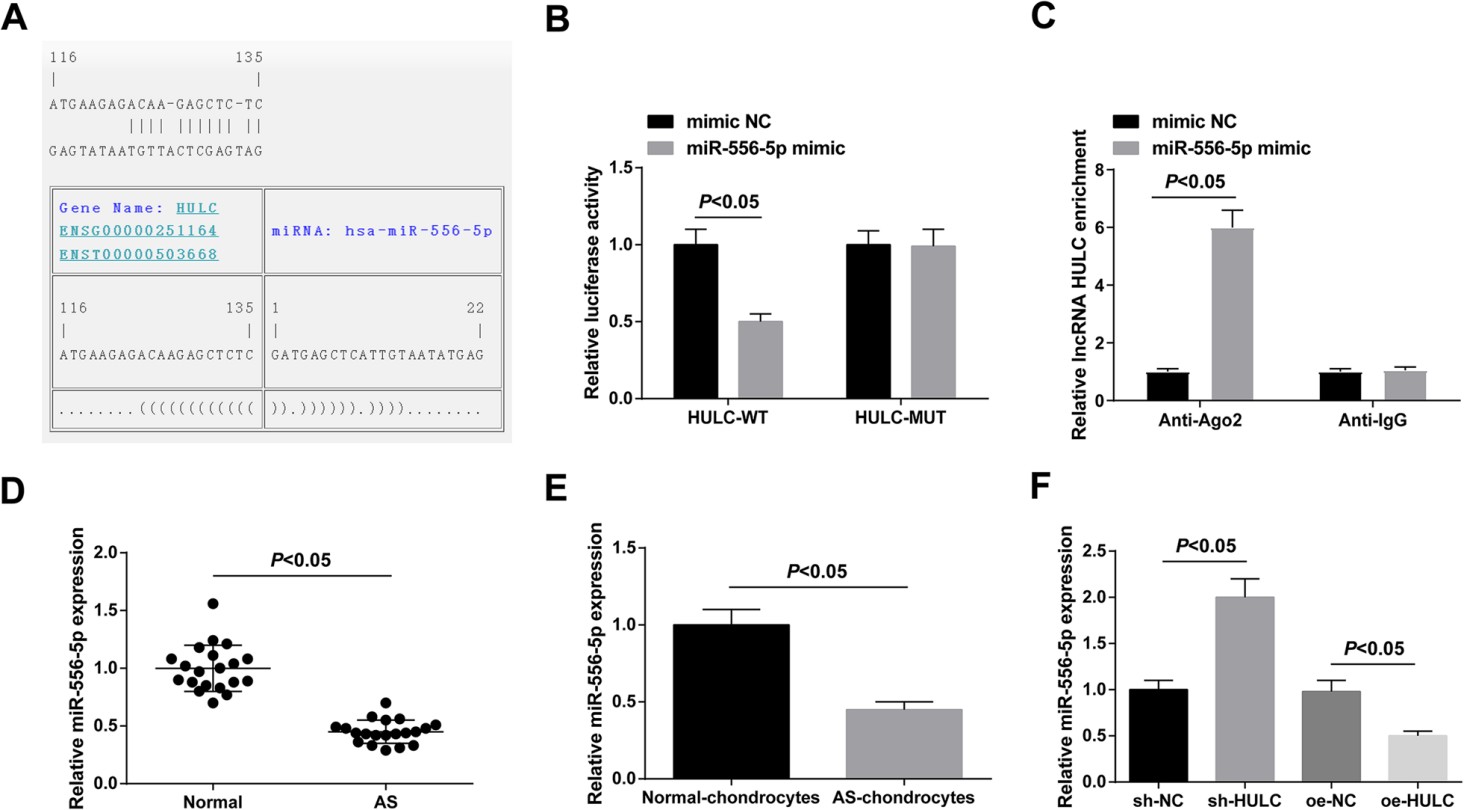
3 miR-556-5p is a downstream factor of HULC. Binding sites of HULC and miR-556-5p predicted by RNA22 database (**A**). Verification of the targeting relationship between HULC and miR-556-5p (**B**). RIP assay was carried out to identify the correlation between HULC and miR-556-5p (**C**). miR-556-5p expression in AS cartilage tissues and chondrocytes (**D**, **E**). miR-556-5p expression in AS chondrocytes after the intervention of HULC expression (**F**). Values were expressed as mean ± standard deviation, *P* < 0.05

The binding site between HULC and miR-556-5p was predicted by RNA22 (Fig. 3A), and the targeting relationship between HULC and miR-556-5p was further verified by dual luciferase reporter gene detection, as evidenced by the results that the luciferase activity was blocked after co-transfection of HULC-WT and miR-556-5p mimic (Fig. 3B). In addition, we took RIP assay to illuminate the endogenous relationship between HULC and miR-556-5p. As suggested in Fig. 3C, HULC were specifically enriched in the Ago2 pellet compared with control IgG immunoprecipitates. miR-556-5p was downregulated in AS cartilage tissues and chondrocytes (Fig. 3D, E), and could be negatively altered in AS chondrocytes after HULC intervention (Fig. 3F).

### Induction of miR-556-5p protects chondrocytes from AS-induced damages

**Fig. 4:**
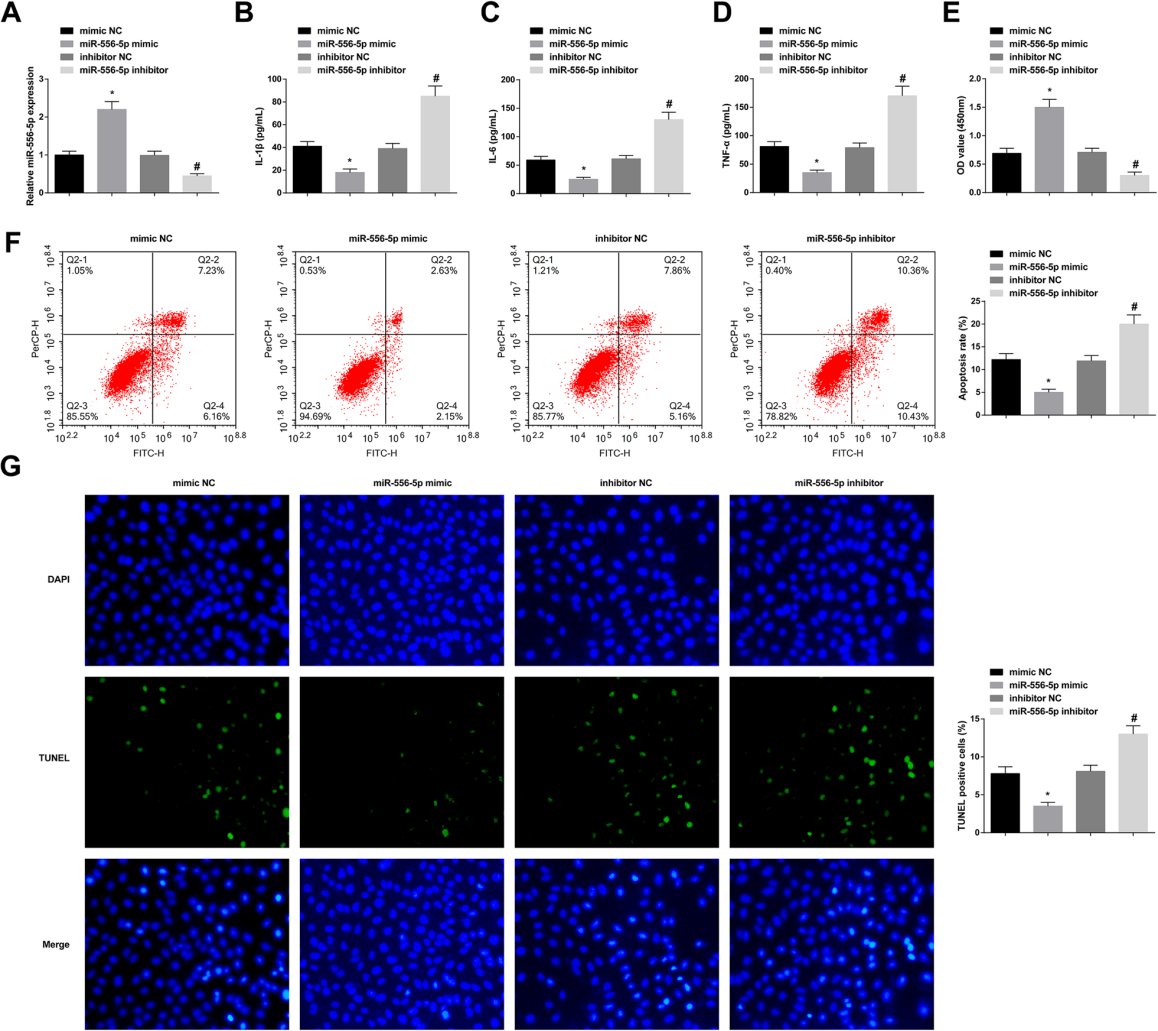
Induction of miR-556-5p protects chondrocytes from AS-induced damages. Successful cell transfection to alter miR-556-5p expression in AS chondrocytes (**A**). After transfection, measurements of IL-1β, IL-6 and TNF-α levels (**B**–**D**), proliferation (**E**), and apoptosis (**F**, **G**). Values are expressed as mean ± standard deviation. *P < 0.05 versus mimic NC; #*P* < 0.05 versus inhibitor NC

RT-qPCR reported the increase in miR-556-5p expression after miR-556-5p mimic treatment, and the expression decreased after miR-556-5p inhibitor treatment in AS chondrocytes (Fig. 4A). Decreased contents of inflammatory factors and enhanced proliferation, in concert with reduced apoptosis, were observable in miR-556-5p-overexpressed AS chondrocytes, while miR-556-5p-underexpressed AS chondrocytes manifested the opposite trend (Fig. 4B–G).

### YAP1 is a potential target gene of miR-556-5p

**Fig. 5:**
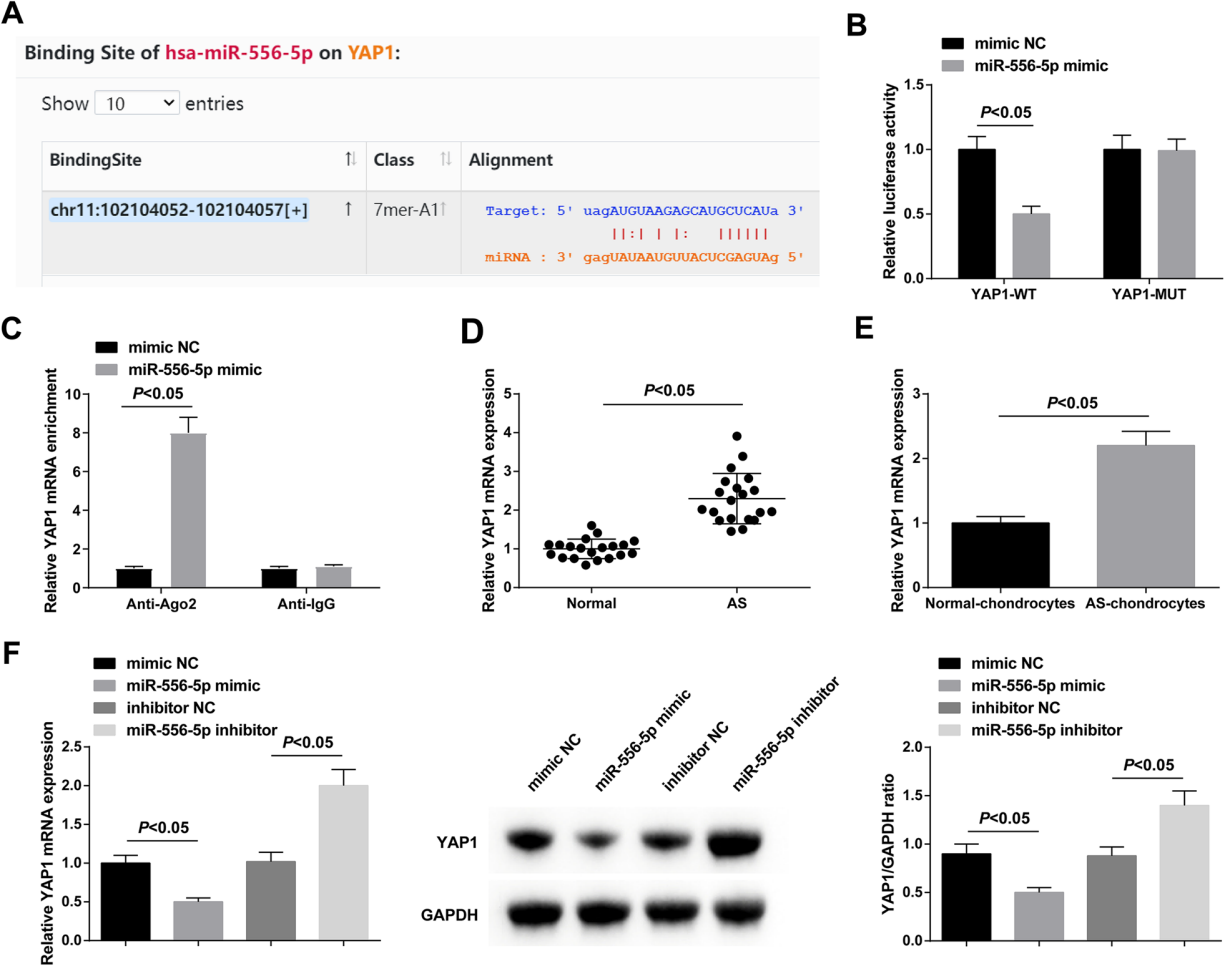
YAP1 is a potential target gene of miR-556-5p. Binding sites of miR-556-5p and YAP1 predicted by starBase (**A**). Verification of the targeting relationship between miR-556-5p and YAP1 (**B**). RIP assay was carried out to identify the correlation between miR-556-5p and YAP1 (**C**). YAP1 expression in AS cartilage tissues and chondrocytes (**D**, **E**). YAP1 expression in AS chondrocytes after the intervention of HULC expression (**F**). Values were expressed as mean ± standard deviation, *P* < 0.05

The binding site between miR-556-5p and YAP1 was identified by starBase prediction (Fig. 5A), and further verification by dual luciferase assay revealed that the luciferase activity was reduced after co-transfection of YAP1-WT and miR-556-5p mimic (Fig. 5B). In addition, we took RIP assay to illuminate the endogenous relationship between miR-556-5p and YAP1. As suggested in Fig. 5C, YAP1 were specifically enriched in the Ago2 pellet compared with control IgG immunoprecipitates. YAP1 levels were enhanced in AS cartilage tissues and chondrocytes (Fig. 5D, E). After elevating miR-556-5p, a reduction was recognized in YAP1 expression, whereas the expression increased after downregulating miR-556-5p (Fig. 5F).

### YAP1 overexpression counteracts silenced HULC-induced protection against AS

**Fig. 6:**
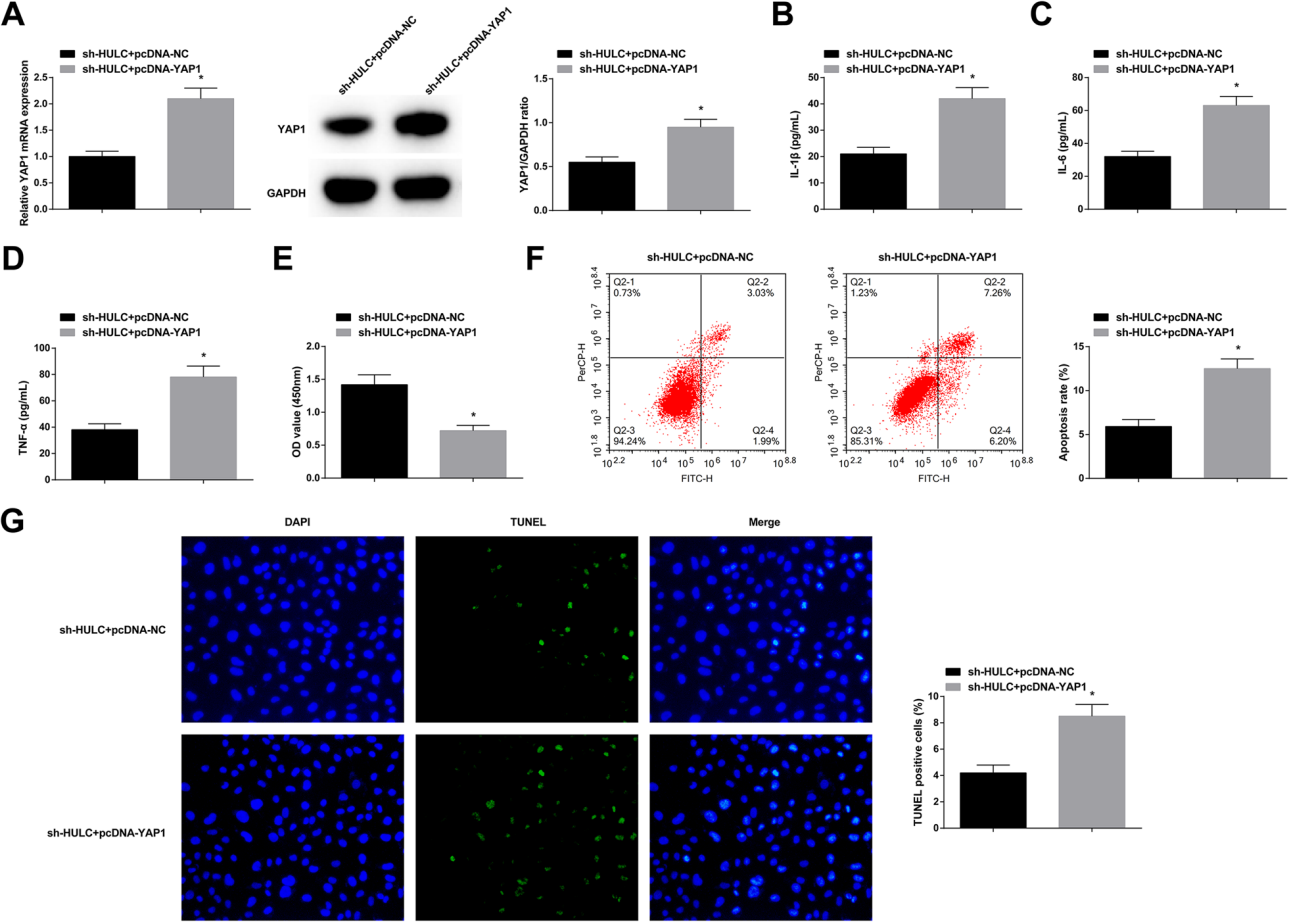
YAP1 overexpression counteracts silenced HULC-induced protection against AS. Successful co-transfection to alter YAP1 expression in AS chondrocytes (**A**). After transfection, measurements of IL-1β, IL-6 and TNF-α levels (**B**–**D**), proliferation (**E**), and apoptosis (**F**–**G**). Values are expressed as mean ± standard deviation. *P < 0.05 versus sh-HULC + pcDNA-NC

The successful transfection of sh-HULC + pcDNA-YAP1 or sh-HULC + pcDNA-NC was verified by RT-qPCR and Western blot (Fig. 6A). Due to YAP1 high expression, the protective actions of HULC silence against inflammation, anti-proliferation, and apoptosis of AS chondrocytes were mitigated (Fig. 6B–G).

### HULC affects the pathological injury of spinal cartilage in AS mice

**Fig. 7:**
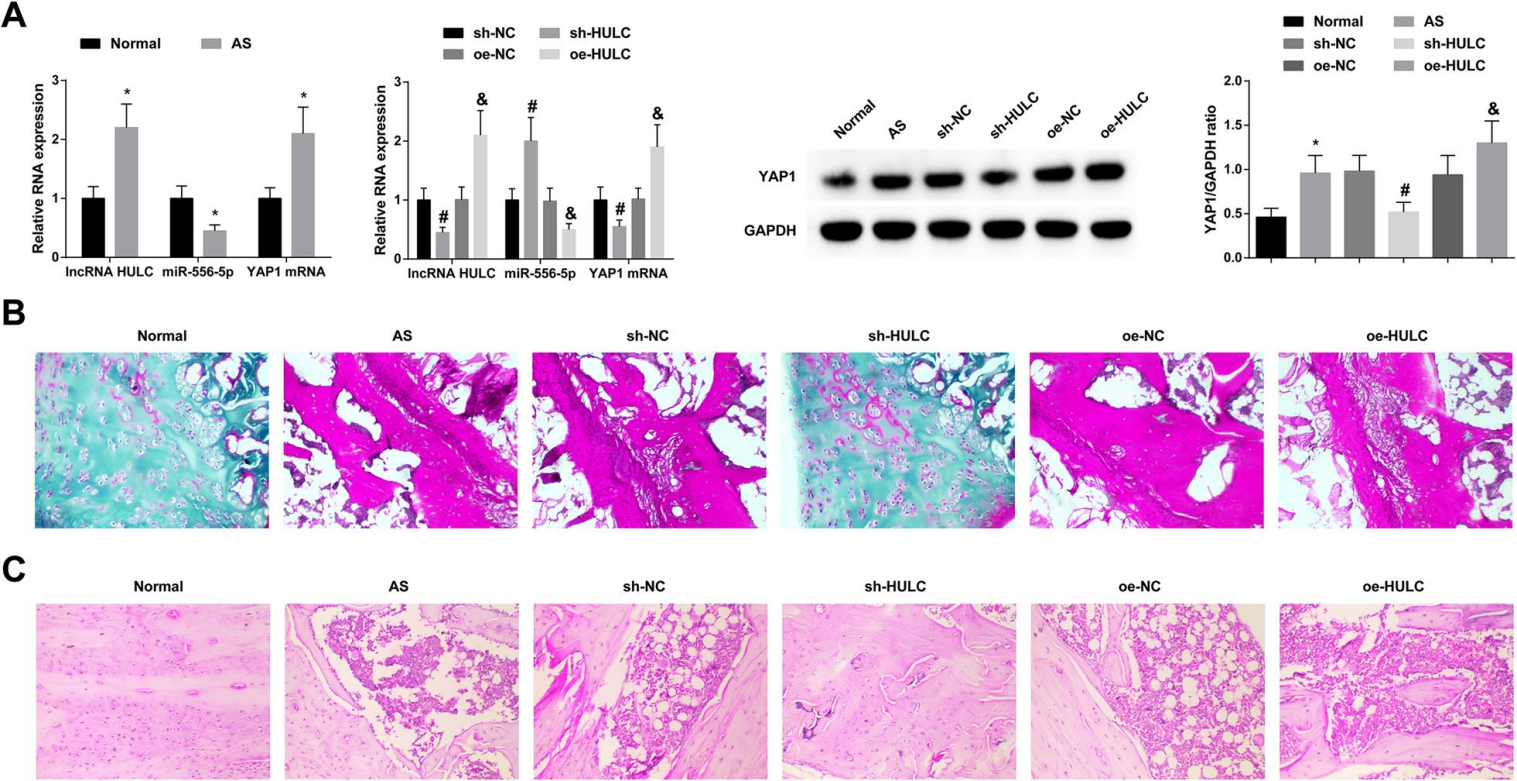
HULC affects the pathological injury of spinal cartilage in AS mice. HULC, miR-556-5p, and YAP1 levels in spinal cartilage in AS mice (**A**). Safranin O staining and HE staining results (**B**, **C**). * *P* < 0.05 versus Normal; #*P* < 0.05 versus sh-NC; &*P* < 0.05 versus oe-NC

In the spinal cartilage of AS mice, high HULC and YAP1, and low miR-556-5p could be measured. After injection with sh-HULC, miR-556-5p levels were restored and YAP1 levels were impaired, while injection of oe-HULC showed the opposite result (Fig. 7A). Then, Safranin O staining and HE staining observed the pathological changes of spinal cartilage. The normal mice had only a few inflammatory cells and fibrocytes impregnated, and the histomorphology was normal without eosinophils. Infiltration of inflammatory cells, fibers, and eosinophils appeared, accompanied by synovial cell proliferation, cartilage tissue infiltration, cartilage tissue transformation and fibrosis, and severely damaged articular surface of cartilage in AS mice. Depleting HULC improved cartilage histopathological injury, while elevating HULC had the opposite effect (Fig. 7B, C).

## Discussion

AS is a complex inflammatory disease with no clear mechanism. Molecular biology and genomics have highlighted the regulatory element of lncRNAs in AS. In AS, lncRNAs are abnormally expressed in different tissues or cells (including hip ligament, whole blood, PBMC, T cells, and osteogenic MSC), and are involved in the regulation of inflammatory signals [3, 24, 26]. This study focused on HULC in AS inflammation and proved that silencing HULC targeted YAP1 through absorption of miR-556-5p, and then inhibited the inflammatory response of AS.

HULC with a length of about 500 nucleotides, mainly exists in the cytoplasm and interacts with ribosomes [40]. HULC is highly expressed in liver cancer and colorectal cancer, which promotes cancer development [40, 41]. Wang et al. [28, 42] have screened out HULC with upregulated expression that may be involved in inflammatory responses. Here, the study measured high HULC expression in AS cartilage tissues and cells, and confirmed that HULC knockdown promoted proliferation, inhibited apoptosis and inflammatory response of AS chondrocytes, and improved the pathological injury of spinal cartilage tissue in AS mice, while HULC induction had the opposite effect.

HULC can act as ceRNA to downregulate miRNAs in disease progression. For example, HULC mediates miR-2052 or miR-372 to affect liver cancer development [43, 44]. HULC can sponge miR-372/miR-373 to influence the malignant activities of cholangiocarcinoma cells [28]. Similarly, this study confirmed that miR-556-5p is the downstream miRNA of HULC. miR-556-5p has been discovered to regulate inflammatory processes of various diseases, such AS systemic lupus erythematosus [45], allergic rhinitis [36], and atherosclerosis [37]. This paper determined that elevating miR-556-5p stimulated chondrocyte proliferation, and blocked apoptosis and inflammation, while silencing miR-556-5p had the opposite effect. It has been previously noted that YAP1 is involved in regulating tissue inflammation. Some reports have measured the upregulation of YAP1 in tumors, such as gastric cancer, colorectal cancer, and pancreatic cancer [46–48]. Subsequently, YAP1 was predicted as the potential target of miR-556-5p, which is consistent with a previous report [49]. Functional assays performed supported that YAP1 overexpression rescued the effects of silencing HULC on AS chondrocytes.

## Conclusion

HULC is high-expressed in AS, which can mediate miR-556-5p to target YAP1, thereby promoting inflammatory responses in AS. HULC knockdown can inhibit the inflammatory response of AS and improve the pathological injury of cartilage tissue. Therefore, HULC is of potential feasibility as a clinical therapeutic target for AS. Considering the limitation of the study, the sample size needs to be enlarged to confirm our conclusions and reduce conclusion bias.

## Data Availability

Data are available from the corresponding author on request.
